# Supplementary material for: End Sequence Analysis Toolkit (ESAT) expands the extractable information from single-cell RNA-seq data
Source: Genome Res. 2016 Oct;26(10):1397–410. doi: 10.1101/gr.207902.116 (PMC5052061; doi:10.1101/gr.207902.116)
Supplement: Supplemental Material [file supp_gr.207902.116_Supp_Materials.tar.gz › ESAT-esat_v0.1/doc/html/classbroad_1_1pda_1_1rnai_1_1_r_n_ai_gene_annotation-members.html]

ESAT: Member List


ESAT


- Main Page
- Related Pages
- Packages
- Classes
- Files

- Class List
- Class Index
- Class Hierarchy
- Class Members

All Classes Namespaces Files Functions Variables Enumerator Pages

- broad
- pda
- rnai
- RNAiGeneAnnotation

broad.pda.rnai.RNAiGeneAnnotation Member List

This is the complete list of members for broad.pda.rnai.RNAiGeneAnnotation, including all inherited members.

|  |  |  |
| --- | --- | --- |
| getGeneName() | broad.pda.rnai.RNAiGeneAnnotation |  |
| getRegion() | broad.pda.rnai.RNAiGeneAnnotation |  |
| getSequence() | broad.pda.rnai.RNAiGeneAnnotation |  |
| getTranscriptName() | broad.pda.rnai.RNAiGeneAnnotation |  |
| getTranscriptSequence() | broad.pda.rnai.RNAiGeneAnnotation |  |
| nanostringHeader | broad.pda.rnai.RNAiGeneAnnotation | static |
| RNAiGeneAnnotation(Gene gene, Alignments linc, String geneName, int versionNumber, String seq, String species, String ncbiBuild) | broad.pda.rnai.RNAiGeneAnnotation |  |
| RNAiGeneAnnotation(String rnaiString) | broad.pda.rnai.RNAiGeneAnnotation |  |
| toNanostring() | broad.pda.rnai.RNAiGeneAnnotation |  |
| toRNAi() | broad.pda.rnai.RNAiGeneAnnotation |  |
| toString() | broad.pda.rnai.RNAiGeneAnnotation |  |


---

Generated on Fri Aug 15 2014 15:11:01 for ESAT by  

 1.8.7
